# Spatial patterns of water-dispersed seed deposition along stream riparian gradients

**DOI:** 10.1371/journal.pone.0185247

**Published:** 2017-09-28

**Authors:** Rob G. A. Fraaije, Sophie Moinier, Iris van Gogh, Robert Timmers, Joost J. van Deelen, Jos T. A. Verhoeven, Merel B. Soons

**Affiliations:** Ecology & Biodiversity Group, Institute of Environmental Biology, Utrecht University, Padualaan 8, CH Utrecht, the Netherlands; Ecole Pratique des Hautes Etudes, FRANCE

## Abstract

Riparian ecosystems along streams naturally harbour a high plant diversity with many increasingly endangered species. In our current heavily modified and fragmented catchments, many of these species are sensitive to dispersal limitation. Better understanding of riparian plant dispersal pathways is required to predict species (re-)colonization potential and improve success rates of stream and riparian zone conservation and restoration. Dispersal by water (hydrochory) is an important mechanism for longitudinal and lateral dispersal of riparian species. Crucially for recruitment potential, it also influences the elevation along the riparian hydrological gradient where seeds become deposited. Due to the complex interplay between abiotic and biotic factors, however, it remains unclear how exactly patterns in seed deposition are formed. We compared hydrochorous and non-hydrochorous seed deposition, and quantified patterns of seed deposition along the bare substrate of newly created stream riparian gradients. Water levels were monitored and seed deposition was measured with seed traps along the full range of riparian hydrological conditions (from permanently flooded to never flooded). Average seed numbers and species richness were significantly higher in flooded than in non-flooded seed traps (5.7 and 1.5 times higher, respectively). Community-weighted trait means indicated that typically water-dispersed seeds were more dominant in flooded than in non-flooded seed traps and gradually decreased in concentration from the channel to the upland. Moreover, highly buoyant seeds accumulated at the average water line, and clear elevational sorting of non-buoyant seeds occurred within the floodplain. These results establish a critical role of flooding in shaping patterns of seed deposition along the riparian gradient, delivering many seeds of typical riparian species to riparian zones and depositing them at species-specific elevations as influenced by seed traits, suggesting species-specific dispersal pathways. This shows that hydrochory likely has important consequences for riparian vegetation development and that flooding forms a key process for successful restoration.

## Introduction

Plant diversity is threatened across the globe by habitat loss and deterioration [1]. In response, restoration projects are carried out to counteract species losses. Unfortunately, successful restoration of abiotic site conditions does frequently not result in re-establishment of desired species [2–4]. Re-colonization by plant species may be hampered either by some (unknown) abiotic or biotic site condition, or simply because species are not able to reach the restored site. The latter is often due to a lack of nearby source populations [5–7], limited dispersal opportunities, and/or a mismatch in spatial patterns of deposited seeds hampering seeds in reaching certain microhabitats [8–11]. A better understanding of plant dispersal pathways is required to be able to predict species (re-)colonization potential and to improve success rates of restoration activities. This applies particularly to riparian ecosystems along streams and rivers, which are among the most heavily degraded ecosystems [12] and among the most frequently restored [13–15].

Riparian plant species may be dispersed by several vectors, including wind (anemochory), animals (zoochory), and water (hydrochory) [16–18]. Hydrochory has been shown to be a dominant dispersal vector in riparian habitats, both in models [18] and in the field: many seeds of many species are dispersed by water to riparian zones [19]. Hydrochory has been shown to bring new species into existing vegetation [20] and to be important for colonization of newly created river banks [21,22]. Yet, recent studies also reported serious constraints in hydrochorous dispersal due to loss of connectivity by damming (longitudinal connectivity), channelization (lateral connectivity) and flow regulation [6,7,17,23].

Seed dispersal via hydrochory is influenced by extrinsic factors (e.g., stream network shape, discharge patterns, connectivity and channel roughness) and intrinsic factors (e.g., seed buoyancy, seed shape and timing of seed release; [17]). Besides determining longitudinal dispersal distance, these factors also determine the position on the stream bank where seeds become deposited. For example, non-buoyant seeds tend to sink to the stream bed, but may be propelled to the floodplain by currents during high flows [24]. Subsequently, spatial sorting may take place by different seed settling velocities in analogy to the deposition of mineral sediments [25,26]. Buoyant seeds, on the other hand, are likely to be spread out across riparian zones following fluctuating water levels [27,28], where they may be concentrated when water levels are stable, or when water levels peak (visible as drift piles that mark high water lines; [29,30]). The eventual position along the riparian gradient has important consequences for the fate of deposited seeds, as hydrological gradients represent a strong environmental filter on recruitment [31], and resulting vegetation patterns and diversity [22,32].

As the qualitative (suitability of deposition site) aspects of hydrochorous dispersal are at least as relevant as the more often studied quantitative aspects (distance), closer inspection of the elevational patterns of seed deposition in relation to seed and species traits may reveal important mechanisms regulating (re-)colonization. Field studies that relate seed traits to elevational patterns of deposition along riparian zones are limited and were mostly carried out in developed plant communities [20,33–35], in which direct seed fall from standing vegetation is likely to dominate deposition patterns. In newly created riparian zones, which are cleared of vegetation, a primary successional stage occurs in which direct seed fall from standing vegetation is still strongly reduced. This provides an ideal environment for collecting information on hydrochorous seed deposition along riparian gradients and its consequences for (re-)colonization and potential restoration success.

We monitored year-round seed deposition in relation to water levels of newly created riparian zones to 1) assess the relative importance of hydrochory in comparison to non-hydrochorous seed delivery to restored riparian zones, 2) improve mechanistic understanding of elevational patterns of seed deposition along riparian gradients and 3) disentangle the effects of seasonality on 1 & 2. To this purpose, we monitored the natural seed rain along riparian gradients of lowland streams that had recently been excavated to bare substrate in the context of a restoration programme. By placing seed traps along a complete range of hydrological conditions (from permanently flooded to never flooded) we could compare hydrochorous dispersal to non-hydrochorous dispersal, and study the effect of hydrochory on elevational seed deposition patterns along riparian gradients.

## Material and methods

### Study system

Stream riparian zones form the boundary between terrestrial and aquatic ecosystems. They are highly heterogeneous and dynamic, characterized by sharp environmental gradients and natural disturbance regimes by flooding [36,37]. Their natural connectivity is high, following propagule transport by anemochory, zoochory and particularly hydrochory, one of the prerequisites for the commonly observed high biodiversity along stream riparian zones. Lowland streams differ from other stream types by their more gentle slope (0–5‰) and low flow velocities (0.05–0.6 m s^-1^), with discharge patterns and associated flooding of riparian zones closely connected to precipitation patterns [13]. In the Netherlands, lowland streams occur directly upstream of the Rhine-Meuse delta, on soils dominantly consisting of sand (aeolian sand deposits) [38]. Most streams in the Netherlands have been degraded by channelization in the last century. The associated disappearance of riparian wetlands has caused severe fragmentation of their remnants. To counteract species and habitat loss, an increasing number of restoration projects has been carried out in the past decades.

We studied three lowland streams in the Netherlands: the Hagmolenbeek (HM), Hooge Raam (HR) and Kleine Aa (KA) (52°13'0.33" N, 6°43'16.88" E; 51°42'57.65" N, 5°42'9.25" E; and 51°35'39.92" N, 5°16'38.71" E respectively). All streams had been subject to restoration measures along 0.8–2 km stream length between 2009 and 2011. Restoration involved the creation of a new channel with a raised stream bed and a narrowed channel to increase flow velocities and lift the groundwater table. This was combined with excavation and widening of riparian zones to create space for inundation and recreate a wide gradually sloping riparian gradient. All pre-existing vegetation was removed upon construction. At HM only, the riparian zone was sown with seeds of *Lolium perenne*, *Trifolium repens*, and *Phleum pratense* subsp. *Pratense* (species nomenclature; [39]) to minimize erosion quickly after restoration. Details on the research locations are available in S1 File (Paragraph S1.1). Permissions to conduct field studies at these locations were issued by private owner Marwin Hofstede and the water boards ‘Aa en Maas’, ‘De Dommel’ and ‘Vechtstromen’.

### Field methods

**Fig 1:**
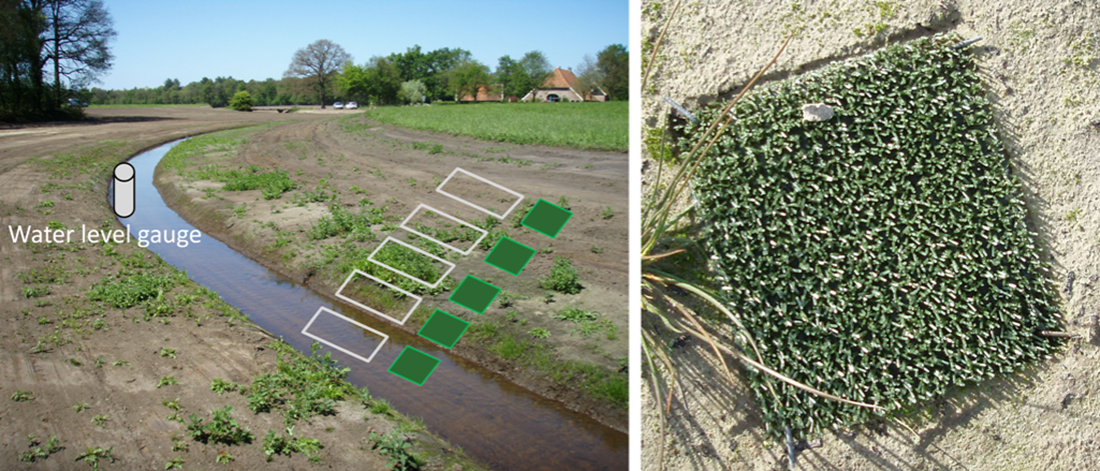
Field study design. To monitor seed rain along the entire hydrological gradient, three replicate transects of five seed traps (squares picture left; close-up picture right) were pinned to the ground perpendicular to the stream channel. Vegetation surveys were carried out in permanent quadrats adjacent to each seed trap (rectangles; picture left). Stream water levels were registered using pressure transducers in water level gauges within 30 m of the nearest transect.

**Fig 2:**
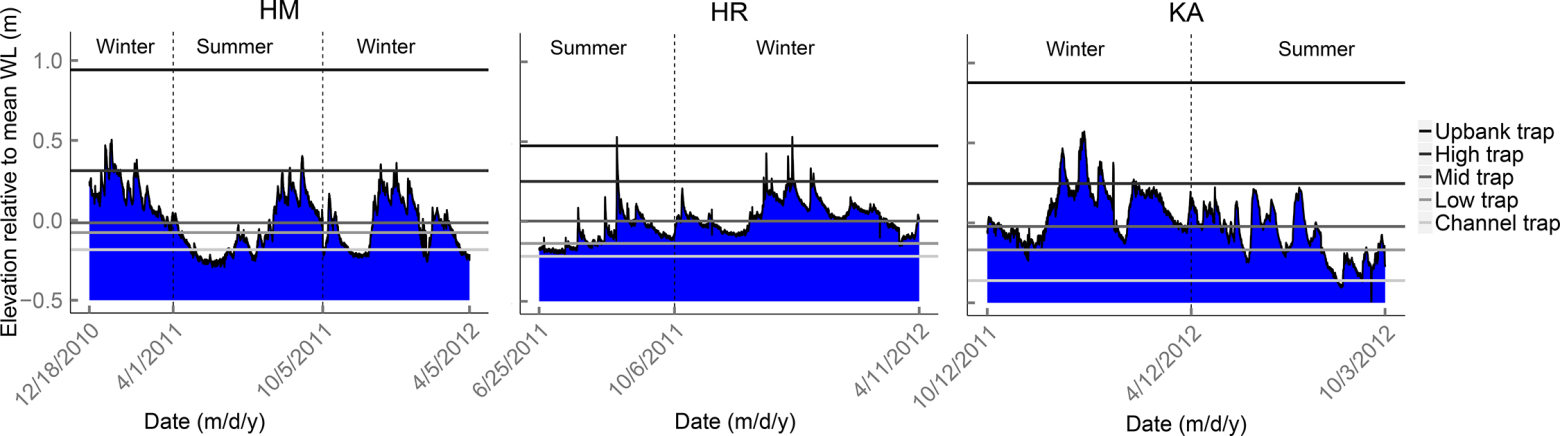
Water level fluctuations relative to the mean water level during the sampling periods for the Hagmolenbeek (HM), Hooge Raam (HR) and Kleine Aa (KA). Sampling periods are separated by vertical dotted lines. Seed trap elevation (relative to mean water level) of representative transects are indicated by horizontal lines.

Natural seed rain was monitored using seed traps made by 25 x 25 cm artificial turf mats (Astroturf, with 1.5 cm bristles and ca. 8 bristles per cm^2^; [34]). To study seed rain along the entire hydrological gradient, three replicate transects of five seed traps were pinned to the ground perpendicular to the stream channel (Fig 1). Distances between seed traps were approximately 0.5, 1.0, 3.0 and 5.0 m (from low to high elevation), and transects were 20–25 m apart, which was the maximum possible in the available area. Seed traps were placed in the field immediately after restoration (HM: December 2010, HR: May 2011 and KA: October 2011) and replaced by new seed traps in April and October to analyse seasonal differences in seed deposition. Seed deposition was monitored in the first year (HR and KA) or first 1.5 year (HM) after restoration. To identify seeds that were deposited by direct seed fall, i.e. seeds that were dropped directly into a seed trap from overhanging or closely surrounding established plant species, vegetation development (species percent cover) was registered in permanent quadrats (25 x 50 cm) adjacent to each seed trap in July/August each year. Stream water levels were registered hourly (Fig 2) using pressure transducers (Schlumberger Water Services, Delft, the Netherlands; Keller Meettechniek B.V., Reeuwijk, the Netherlands) in water level gauges within 30 m of the nearest transect.

### Lab methods

After each trapping period, seed traps were taken to the laboratory and stored in plastic bags at 4°C in the dark before processing. Processing involved the extraction of deposited material by flushing the seed traps with water, followed by wet-sieving the deposits with three sieves of 2.0, 0.5 and 0.1 mm in order to remove fine silt and clay and group seeds of similar size together. In the next step, deposited material of the Hagmolenbeek seed traps was dried at 70°C for 48 hours, after which seeds were extracted by hand and identified to species level using dissecting microscopes and a seed identification guide [40]. This method was very time-consuming and therefore seedling emergence trials were used to count and identify seeds in the seed traps of the remaining sites [20,21,41]. This procedure involved spreading out the wet-sieved deposited material over waterlogged sand-filled trays (60 x 40 x 10 cm) in a greenhouse facility with a 15:9 h light:dark period (maintained by 400W overhead plant growth lights) and an air temperature between 15 and 25°C [41]. To provide favourable germination conditions for as many species as possible, the trays remained waterlogged in the first 10 weeks, were slowly dried out in the following 4 weeks, and were then submerged by a 5-cm water layer for 4 weeks. Emerging seedlings were removed weekly against overcrowding and were identified to species level (species nomenclature; [39]).

The different seed count methodologies resulted in similar amounts of species. Although the total number of seeds was higher for physical extraction, this difference was largely caused by seeds of only three highly abundant species. This more likely reflected differences between research locations than between seed count methods as further detailed in S1 File (Paragraph S1.2). Overall, this gave us no reason to expect that the different methods used affected the study results.

### Plant and seed traits

To identify the type of species transported through hydrochory, and the mechanisms that shape patterns of seed deposition along riparian gradients, we analysed plant and seed traits related to hydrochory and/or expected to influence elevational patterns of seed deposition, retrieved from the LEDA trait database [42,43]. These included 1) species’ reliance on hydrochorous dispersal; proportion of hydrochory in all assigned dispersal modes of a species, choosing from hydrochory, anemochory, zoochory, autochory, hemerochory (dispersal by man), and ‘other’ (tumbleweeds and raindrop-ballists), 2) seed buoyancy; percentage of seeds still floating after 1 week in water and 3) seed specific weight; seed mass divided by volume, assuming an ellipsoid seed shape with volume = 4/3 x pi x 0.5l x 0.5w x 0.5h for all seed species, an important determinant of settling velocity in the water column [25]. To identify potential consequences of seed deposition patterns for vegetation development we analyzed species moisture requirements as indicated by Ellenberg F-values ([44], adjusted by [45], which have been demonstrated to be strong predictors for recruitment patterns along hydrological gradients [31].

### Data analysis

The newly created riparian zones were devoid of vegetation at the beginning of seed rain monitoring. However, vegetation became established during the monitoring period which contributed seeds to the trapped seed pools by direct seed fall (seeds from standing vegetation dropping directly into a nearby seed trap). To analyze hydrochorous seed deposition patterns along riparian gradients without distortion to these patterns by fall from standing vegetation we excluded from our seed trap data any seeds of species that already became established in a transect in the period of seed rain monitoring.

To evaluate the importance of hydrochory relative to non-hydrochorous seed dispersal we compared seed deposition in flooded versus non-flooded seed traps during the monitoring period. In this analysis only data of the HM and KA sites were used (there were no non-flooded seed traps at the HR-site). To analyze spatial patterns in seed deposition along the riparian gradient we related seed trap position (elevation relative to the mean stream water level during seed rain monitoring) to seed deposition. The relative elevation to mean water level has previously been used as a useful surrogate for otherwise strongly inter-correlated hydrological variables including flooding frequency, flooding duration and mean flooding depth [34]. Analysis of seed deposition included the number of species, number of seeds, community-weighted trait means (abundance weighting as recommended for species-poor habitats like several seed traps in our dataset; [46–48]), and community composition.

For analyzing the number of species and number of seeds we used generalized linear mixed models with a negative binomial error distribution (R package lme4; [49]). For analyzing community-weighted trait means we used linear mixed models and the same R package (with log- or arcsine-transformation of response values to improve normality when necessary, based on the Shapiro-Wilk test). Transects and time of monitoring after restoration (either the first, second, or–only for HM–third half year after restoration) were used as random effects (intercept) in all models. The effect of season, and either flooding (categorical variable: flooded or non-flooded) or seed trap position along the riparian gradient were added as fixed effects. In the models which tested the effect of flooding (on the HM and KA data), time of monitoring was not included as a random effect, as this interfered with the fixed effect of season. Although transects were nested within stream in the study design, streams were not included as an additional random effect. Exploratory analysis revealed that transects captured most variability of the mixed design while obtaining comparable but more stable results. Forward selections were carried out to test significance of fixed effects, with model fits determined by the Akaike’s Information Criterion (AIC). In each forward selection step, a fixed effect yielding the lowest AIC was selected, with at least 2 units decrease in AIC for addition of a fixed effect [50].

Community composition was analyzed using a partial redundancy analysis on Hellinger-transformed species data [51], using the R-package vegan [52]. Hellinger transformation was used to reduce the weight of the most abundant species [53]. Transects were partialled out in all multivariate analyses.

## Results

We retrieved 100 of the 105 seed traps that were placed in the field. Of these traps, 82 experienced flooding during the period of seed rain monitoring and 18 –only from the HM and KA sites–did not (Fig 2; Table 1). Initial seed arrival (seed rain of species that were not yet established in a transect during seed rain monitoring; see Data analysis) was detected for 110 species and 18,842 seeds in total, of which 102 species and 17,816 seeds were deposited in flooded seed traps and 48 species in non-flooded seed traps (1,026 seeds). Although the number of species was generally comparable between sites, the overall numbers of seeds were higher at the HM site. This was largely due to three highly abundant species: *Rorippa palustris* (7,000 seeds), *Gnaphalium uliginosum* (3,028 seeds) and *Betula pendula/pubescens* (2,458 seeds), together accounting for 12,486 deposited seeds. A complete species list is given in S2 File.

**Table 1:** Overview of the number of species and number of seeds deposited in seed traps per research site, period of monitoring and season.

Site	Period	Season	Traps FL/NotFL (#/#)	Sp TOT (#)	Sp FL (#)	Sp NotFL (#)	Seeds TOT (#)	Seeds FL (#)	Seeds NotFL (#)
HM	1	Winter	12/3	26	23	13	8921	8849	72
HM	2	Summer	12/3	22	21	8	3348	3181	167
HM	3	Winter	11/4	69	64	23	3804	3663	141
HR	1	Summer	15/0	27	27	-	380	380	-
HR	2	Winter	15/0	33	33	-	551	551	-
KA	1	Winter	7/3	31	28	15	1080	777	303
KA	2	Summer	10/5	27	16	20	758	415	343
All seed traps			82/18	110	102	48	18842	17816	1026

Numbers of traps, species (Sp) and seeds are given for flooded (FL), not-flooded (Not FL) and all (TOT) monitored seed traps.

### Relative importance of hydrochory for seed arrival

**Fig 3:**
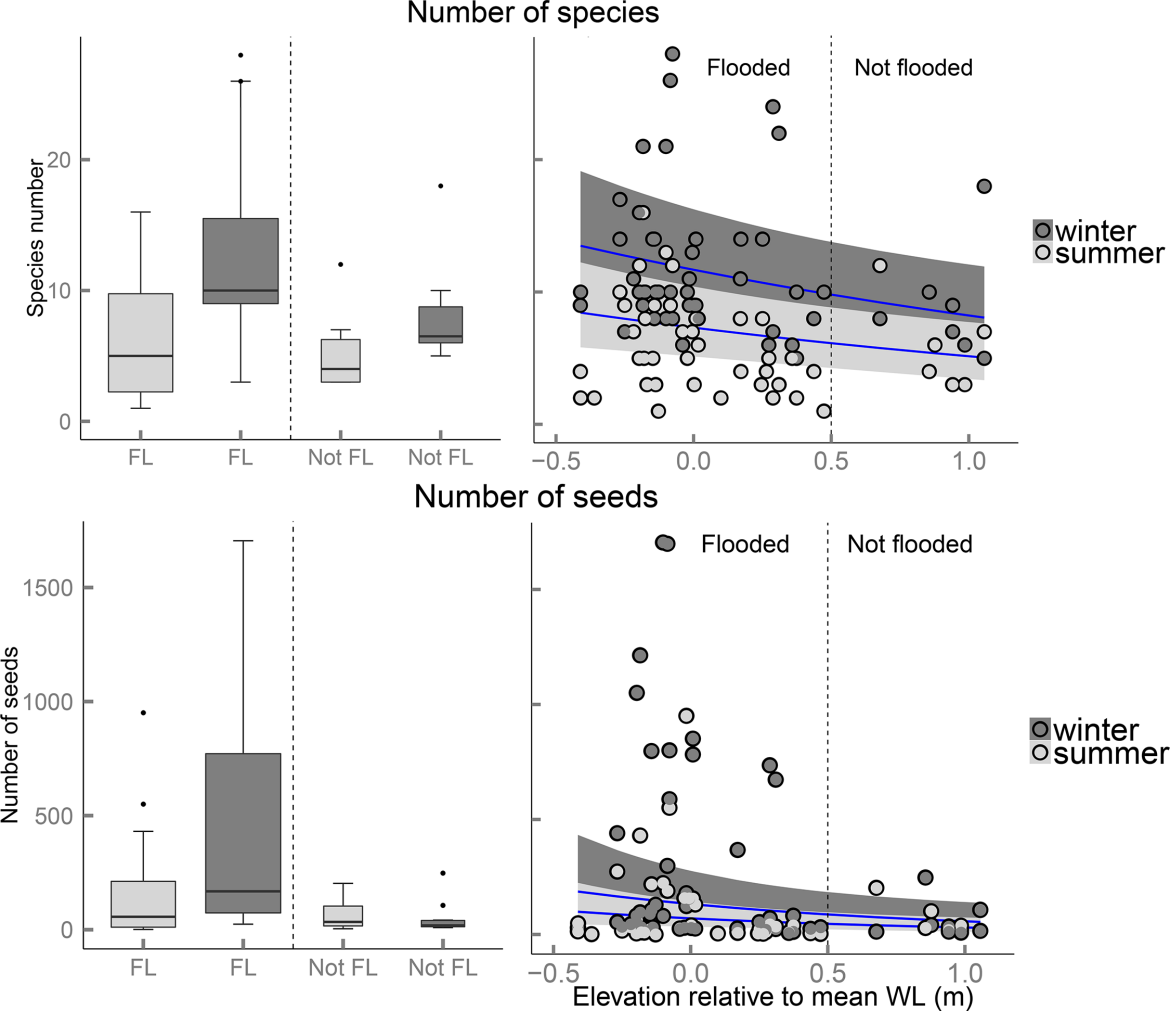
The effects of flooding and season on the number of species of deposited seeds (upper panels) and number of seeds (lower panels). A comparison of flooded (FL) and non-flooded seed traps (Not FL) is given in the left panels (box widths proportional to number of seed traps). The effect of seed trap elevation relative to mean water level is given in the right panels (negative values represent seed traps below the average water level, positive values represent seed traps above it). Summer and winter data are displayed in different greyscales. In the right panels, blue lines represent modelled relationships between field data (circles) and the explanatory variables season and elevation, as analyzed in negative binomial GLMMs. Grey ribbons indicate the 95% confidence intervals (based on fixed effects only).

Hydrochory greatly contributed to seed arrival at the restored riparian zones. Average numbers of species and seeds were significantly higher in flooded seed traps than in non-flooded seed traps (Fig 3 left panels; Table 2). For the number of species, this difference was greatly due to the fact that many species arrived exclusively at flooded seed traps (48 species, contributing 1,216 seeds). For the number of seeds, the difference was mostly generated by species that arrived both in flooded and in non-flooded traps (38 species, contributing 16,674 seeds). Of this latter group, 13 species arrived in much higher numbers at flooded seed traps than at non-flooded seed traps (14,427 versus 159 seeds; see S3 File).

**Table 2:** Forward selections of fixed effects in (negative binomial G)LMMs explaining species number and number of seeds in the seed traps.

		# Species		# Seeds		Reliance hydr		Buoyancy		Specific weight		Ellenberg F	
Flooded versus non-flooded													
	**Effects**	**R**^**2**^	**AIC**	**R**^**2**^	**AIC**	**R**^**2**^	**AIC**	**R**^**2**^	**AIC**	**R**^**2**^	**AIC**	**R**^**2**^	**AIC**
	None	0	434.2	0	895.0	0	-174.0	0	56.2	0	138.7	0	220.1
	Seas	0.35	417.1	0.15	890.9	0.02	-173.8	0.11	50.3	0.02	139.3	0	222.1
	FL	0.11	432.0	0.44	885.5	0.14	**-182.4**	0.20	42.7	0.06	**136.6**	0.18	**208.4**
	Seas + FL	0.44	**413.6**	0.56	**882.8**	0.16	-182.2	0.30	**34.6**	0.08	136.9	0.18	210.4
	Seas + FL + Seas:FL	0.42	414.5	0.52	881.6	0.15	-180.3	0.30	36.1	0.08	138.8	0.19	210.7
Seed trap elevation (all traps)													
	**Effects**	**R**^**2**^	**AIC**	**R**^**2**^	**AIC**	**R**^**2**^	**AIC**	**R**^**2**^	**AIC**	**R**^**2**^	**AIC**	**R**^**2**^	**AIC**
	None	0	574.8	0	1179.6	0	-266.1	0	73.7	0	188.3	0	327.9
	Seas	0.23	558.6	0.07	1175.4	0.02	-265.9	0.08	67.2	0.04	186.1	0.03	327.7
	Elev	0.08	569.2	0.15	1176.2	0.19	**-291.4**^**E**^	0.12	59.0	0.11	180.8^Q^	0.22	303.9
	Seas + Elev	0.28	**551.2**	0.21	**1171.8**	0.20	-292.5^E^	0.18	**49.0**	0.45	**178.4**^**Q**^	0.24	302.0
	Seas + Elev + Seas:Elev	0.28	553.1	0.23	1172.0	0.20	-290.6^E^	0.18	51.0	0.14	182.2^Q^	0.30	**293.8**^**E**^
Seed trap elevation (only flooded traps)													
	**Effects**	**R**^**2**^	**AIC**	**R**^**2**^	**AIC**	**R**^**2**^	**AIC**	**R**^**2**^	**AIC**	**R**^**2**^	**AIC**	**R**^**2**^	**AIC**
	None	0	474.0	0	975.9	0	-216.2	0	49.1	0	159.2	0	260.0
	Season	0.21	456.8	0.07	969.9	0.02	-216.5	0.07	41.8	0.05	156.4	0.03	259.9
	Elev	0.02	472.0^Q^	019	966.9^Q^	0.12	-227.8	0.04	46.6	0.17	146.8^Q^	0.13	249.4
	Seas + Elev	0.23	453.2^Q^	0.25	**957.3**^**Q**^	0.14	-228.8	0.11	**38.4**	0.22	142.9^Q^	0.15	248.7
	Seas + Elev + Seas:Elev	0.38	**447.2**^**Q**^	0.43	956.7^Q^	0.18	**-231.0**^**Q**^	0.11	40.3	0.28	**137.8**^**Q**^	0.21	**244.1**

Fixed effects included flooding (‘FL’; flooded *versus* non-flooded) and season (‘Seas’; summer *versus* winter) for models above the dashed line. The effects of Elevation relative to mean water level (‘Elev’; exponential or quadratic when significantly better than linear, indicated in superscript with ‘E’ and ‘Q’) and season were tested below the dashed line, separately for all seed traps and for the subset of only flooded seed traps. Explained variance (R^2^, determined from models with a Poisson error distribution) and model fits (AIC) are given for each model. Best models, with at least 2 units AIC decrease for addition of a variable, are underlined.

**Fig 4:**
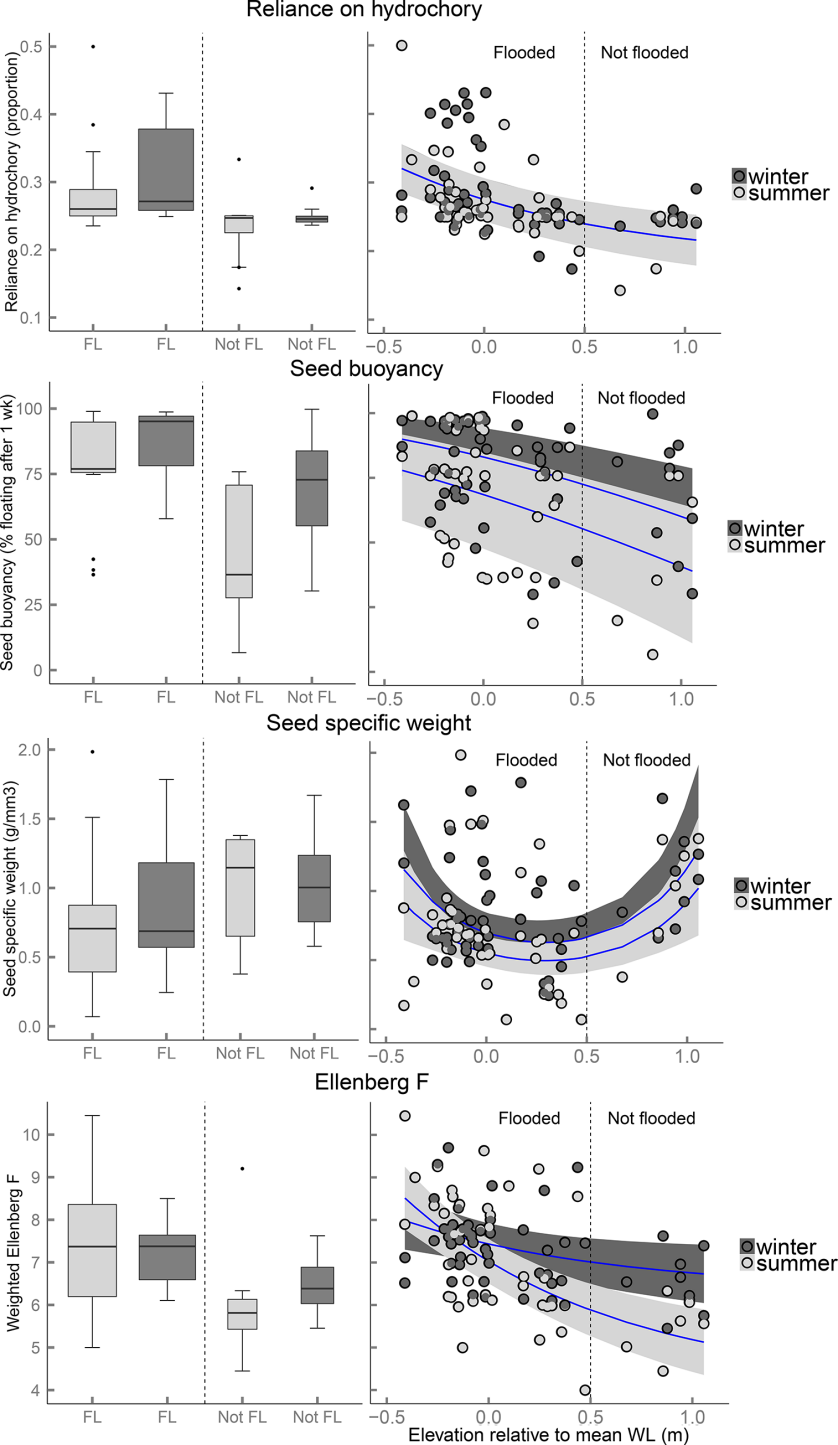
The effects of flooding and season on community-weighted means of seed traits of the deposited seed community. A comparison of flooded (FL) and non-flooded (Not FL) seed traps is given in the left panels (box widths proportional to number of seed traps). The effect of seed trap elevation relative to the mean water level is given in the right panels (negative values represent seed traps below the average water level, positive values represent seed traps above it). Summer and winter data are displayed in different greyscales. In the right panels, blue lines represent modelled relationships between field data (circles) and the explanatory variables season and elevation, as analyzed in LMMs. Grey ribbons indicate the 95% confidence intervals (based on fixed effects only).

These differences in seed deposition between flooded and non-flooded sites shaped differences in the arriving seed communities along the riparian gradient. Multivariate analysis showed that both flooding and season had a significant effect on seed community composition (total adjusted explained variance for HM site: R^2^ = 0.19, P = 0.001, and for KA-site: R^2^ = 0.16, P = 0.001; S4 File (Paragraph S4.1). Community-weighted trait means further showed that seeds deposited in flooded seed traps had a significantly higher reliance on hydrochory, higher seed buoyancy, lower seed specific weight and higher Ellenberg F-values than seeds deposited in non-flooded seed traps (Fig 4 left panels; Table 2).

Apart from flooding, season of arrival explained additional variance. In winter significantly more species and seeds were deposited, with higher community weighted means for buoyancy than in summer (Figs 3 and 4; Table 2).

### Elevational patterns of seed deposition along riparian gradients

Besides differences between flooded and non-flooded seed traps, more gradual patterns in seed deposition were observed along the riparian gradients. The number of seeds and species showed significant gradual decreases from the channel to the upland (Fig 3; right panels). Although both response values showed signs of a peak near the average water level (i.e. zero elevation relative to mean WL), a quadratic term for seed trap elevation (describing a unimodal pattern along the gradient) was not significantly better than the main linear term when analyzing the full data-set. When we excluded the non-flooded seed traps, however, unimodal curves with peaks around the average water level gave the best fit (S5 File).

**Fig 5:**
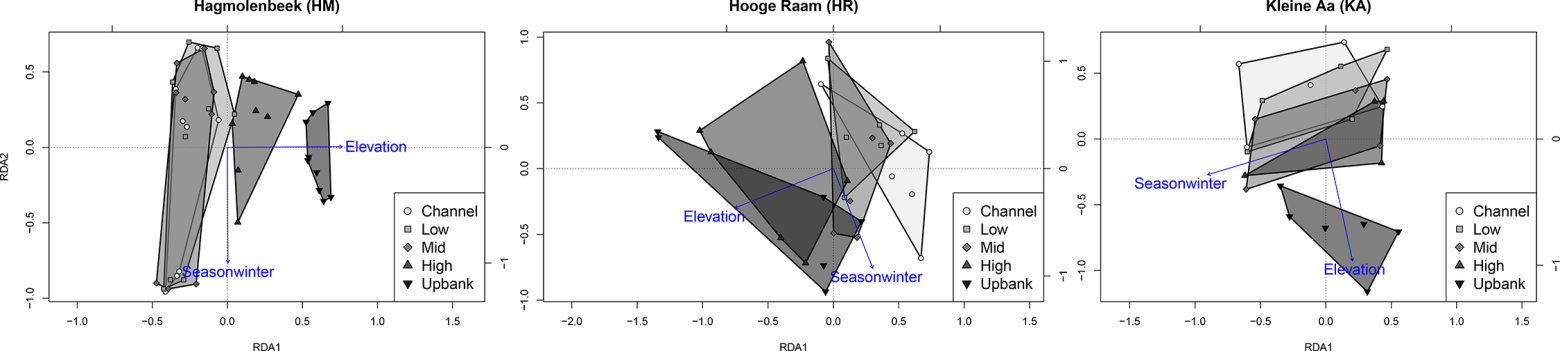
Distance triplots of partial redundancy analyses on Hellinger-transformed seed deposition per research location with respect to the explanatory variables season and seed trap elevation. Site scores are indicated by symbols, with symbol type determined by seed trap position along the riparian gradient, ranging from the dry end (upbank) to the wet end (channel) of the riparian gradient. Contour lines are added around sites scores with the same symbol type.

Community composition of the arriving seeds also showed a clear pattern along the riparian gradient. Multivariate analyses distinctly separated communities from different elevations along the riparian gradient (Fig 5). In these analyses, both seed trap elevation and season were significant explanatory variables (total adjusted explained variation R^2^ = 20.4, 9.8, and 16.8 for HM, HR and KA respectively, with P = 0.001 for all three analyses; S4 File, Paragraph S4.2). This result was also found when only the subset of flooded seed traps was analyzed, except for the KA-site where only the effect of season remained significant (S4 File, Paragraph S4.3).

Further analysis of the deposited seed communities revealed gradual decreases in community-weighted trait means from the channel to the upland for reliance on hydrochory, seed buoyancy and Ellenberg F-value (Fig 4; right panels). For seed specific weight, however, a U-shaped quadratic relation was found (Fig 4). When excluding the non-flooded seed traps, all relations remained more or less similar (Table 2; S5 File), except that seasonal differences were stronger.

Season explained additional variance in nearly all analyses on community patterns along the riparian gradient, with and without non-flooded seed traps in the analyses. In winter a larger number of seeds and species, and higher values for seed buoyancy and seed specific weight were found than in summer. Moreover, in winter higher Ellenberg F-values were observed particularly for seed communities on higher elevations in the floodplain, as visible by a significant interaction between season and Ellenberg F-value (Fig 4; Table 2). When excluding the non-flooded seed traps, more interactions with season were significant. Particularly, in winter the deposition of seeds and species around the average waterline was less pronounced than for the strongly unimodal curves for summer (S5 File).

## Discussion

### Relative importance of hydrochory for seed arrival

Seed deposition following transport by stream water played a very important role in seed arrival at restored lowland stream riparian zones. Both the number of seeds and the number of species were significantly higher in seed traps that were at some point flooded by stream surface water than in non-flooded seed traps. This difference was also reported in studies on seed dispersal across vegetated stream riparian zones [19,20,34] and can be explained by the additive role of hydrochory to other dominant dispersal processes such as anemochory and zoochory. In our study, 48 species were deposited exclusively, and many of the abundant species were deposited dominantly, at flooded seed traps. In accordance with studies on vegetated riparian zones, the effect of hydrochory was particularly strong in winter, adding higher numbers of seeds and species than in summer [19,20].

The community composition of seeds that arrived in flooded seed traps differed from that in non-flooded seed traps, which was also clearly visible in the analyzed species traits. Species arriving in flooded seed traps were more typically water-dispersers (more reliant on hydrochory), had a higher seed buoyancy and a lower seed specific weight than species arriving in non-flooded seed traps. Such traits are typical for riparian plant species: riparian species along rivers have significantly higher seed buoyancies than species from other ecosystem types [54]. Also, the species arriving in flooded seed traps were characteristic of habitats with higher moisture levels (as indicated by their higher Ellenberg F-values), as riparian plant species would be expected to be. One explanation for the large amounts of riparian plant species in the flooded seed traps is the direct vicinity of riparian plant populations to streams, providing ample upstream seed sources. Seeds of these species become dropped directly in the water or on floodplains where they will easily be carried away by surface water during high flows [17]. For the same reason aquatic species might be expected to contribute substantially to hydrochorous propagule pools, but in our study the number of seeds of this species group was low compared to riparian species. This may be caused by the dominantly vegetative regeneration strategies of aquatic species [55,56], but is most certainly also caused by the smaller upstream species pools (in number of individuals) of aquatic species compared to riparian plant species. Such regional factors have been shown to strongly determine the amount and composition of local seed rain in riparian zones [6,57,58]. A second explanation for the large amounts of riparian plant species in the flooded seed traps might be that riparian plants produce long-floating seeds, which are ideally suited to be deposited by the stream water at the shorelines where they prefer to germinate and grow [22]: a form of highly effective ‘directed dispersal’ [59,60].

Despite the high contribution of riparian plants in the flooded traps, a large amount of variation was evident for all seed traits and also many non-typical water-dispersers arrived via hydrochory. This may in part be explained by the regular occurrence of other species than typical riparian plant species in floodplains (e.g., common and/or opportunistic terrestrial species; [61]), of which seeds also easily become part of the hydrochorous seed pool. Additionally, at unrestored stream sections the upland is very close to the stream, which facilitates seeds of upland species to end up in the stream via wind, animals or overland flow after heavy rains [62]. Otherwise, typically wind-dispersed species may travel long distances [16,63] before being ‘trapped’ in a stream [17]. These seeds often float well, resulting in ‘non-standard’ hydrochorous dispersal that may incidentally be effective [64–66].

### Elevational patterns of seed deposition along riparian gradients and explanatory mechanisms

The numbers of seeds and species both decreased along the channel-upland gradient. This suggests that the number of hydrochorously-dispersed seeds decreases with increasing elevation, which was supported by gradual declines in community-weighted means for reliance on hydrochory, seed buoyancy and Ellenberg F-value along the gradient (i.e., seeds becoming less similar to the hydrochorous seed pool with increasing elevation). Within the hydrochorously-deposited seeds, however, also patterns in seed deposition along the riparian gradient occurred. Firstly, the number of seeds and species both showed a peak around the average water line (additionally to the gradual decrease), probably by the deposition of high numbers of floating seeds. Floating seeds may be entrapped in riparian vegetation [67], or get stranded by retreating water levels [28], which frequently occurs along the average water line. Particularly species with long-floating seeds are known to get dominantly deposited around this fluctuating water line [60,68], or at higher elevations in drift lines [30,69]. Unfortunately, the quality of the buoyancy data does not allow us to detect patterns in the deposition of long-floating seeds; the data represent floating percentages after only one week in water, which are not sufficiently representative for long floating times (months, years). Reliance on hydrochory, however, did show a peak around the average water line (in winter), indicating that seeds arriving around the average water line were typical water-dispersers.

Secondly, community-weighted means for seed specific weight increased towards the wet end of the gradient where actually a decrease would have been expected based on the increasing number of hydrochorous seeds (which generally have a lower seed specific weight). Apparently, hydrochorous seeds with a higher specific weight became mainly deposited at lower elevations, while hydrochorous seeds with a lower specific weight became deposited at higher elevations within the flooded range. This pattern resembles spatial sorting in the deposition of non-floating particles. Mineral sediments with different settling velocities [25] have been shown to become deposited at different distances from the stream during flooding [26]. In analogy to this, non-buoyant seeds with high specific weights may become deposited close to the channel, while non-buoyant seeds with lower specific weights end up at further distances (elevations) from the stream. Analogies between the deposition of seeds and that of mineral sediments have been proposed in earlier studies [67,70]. Our data supports this and suggests an important role for seed specific weight in hydrochorous seed sorting across the riparian zone.

Lastly, community-weighted Ellenberg F-values indicated that species with adult optima at wetter conditions dominated seed arrival at lower elevations (channel seed traps), while species with drier optima dominated at higher elevations in the floodplain. This species sorting was stronger than would be expected by a gradual decrease in hydrochorous seeds, indicating that hydrochory brings many seeds to their preferred microhabitats, as proposed earlier by [60]. As mentioned above, seed buoyancy was probably important to this form of directed dispersal, stimulating seeds with a high buoyancy to be deposited around the average water line or higher, while for seeds with a low buoyancy deposition under water is stimulated [71,72]. Although our study does not allow to compare the relative contributions of the mechanisms that shape seed deposition patterns, it clearly indicates that seed buoyancy, seed specific weight and stream surface water (flow and level) in combination are likely to result in seed deposition and species sorting across the riparian zone.

### Seasonal differences

Our data show that seed arrival is higher in winter than in summer, while comparable numbers or even the opposite has been found in existing vegetation where direct fall from standing vegetation may contribute largely to seed deposition ([73], A.G. Garssen, unpubl. data). Specifically for hydrochorous dispersal, however, the most important effect of seasonality is that in winter, more seeds of more species are dispersed, supporting earlier studies [19,20], and that wetland species are more widely distributed over the riparian zone (as indicated by the community-weighted Ellenberg F-values). This underlines the importance of high flows during winter, enabling riparian plant species to colonize a wider range of elevations across riparian zones.

### Consequences of hydrochorous seed dispersal for vegetation development

Our results demonstrate that hydrochory contributes importantly to the arrival of seeds in riparian zones: hydrochorous dispersal results in the arrival of many seeds of many species in riparian zones, potentially contributing to local vegetation diversity [31,32,74,75]. Furthermore, the spatial patterns of seed deposition along the riparian gradient showed that hydrochory generates a species sorting template which may stimulate species coexistence along these gradients, and on which further community processes may operate to generate vegetation patterns [22,31,76,77]. Particularly, hydrochorous dispersal contributes to the arrival of seeds of (common) riparian wetland species, and brings many seeds to their preferred microhabitats, thereby playing an important role in their population dynamics and colonization potential [4,60,78]. Hydrochorous dispersal may further contribute to the dispersal of non-typical riparian species of which many are deposited in riparian zones–although not necessarily at favourable sites. In these ways, hydrochory is likely to have important consequences for riparian vegetation development, which is expected to be particularly strong at recently excavated stream riparian zones. In fully developed vegetation, on the other hand, the contribution of hydrochory to (re-)colonization and gene flow is likely to be lower, as it is more difficult for arriving seeds to germinate and establish in existing plant communities.

The above has several implications for the conservation and restoration of lowland riparian zones, particularly in human-dominated landscapes. Firstly, in increasingly human-dominated landscapes the loss of connectivity between stream sections (due to damming, diversions) and between the stream and its riparian zone (due to channelization and prevention of flooding) currently greatly reduces the potential for hydrochorous dispersal of riparian plant species [17,23]. This ongoing fragmentation increasingly hampers gene flow between existing populations and re-colonization of restored sites (as also shown for mountain streams; [4]). Restoration of connectivity and particularly of flooding seems essential to ensure sufficient propagule arrival to riparian zones. Secondly, clear spatial patterns were observed in the deposition of hydrochorous seeds along the riparian gradients. These patterns may contribute to floristic diversity of riparian zones [22,79], but also emphasise that not all species arrive everywhere along the riparian gradients. This shows that propagule or seedling introduction at specific elevations along riparian gradients may be necessary if establishment of certain species is targeted. Finally, to ensure a quick colonization of restored riparian zones, with a range of typical riparian plant species, restoration activities removing any unwanted existing vegetation should best be finished before winter. With such timing, restoration optimally benefits from the high numbers of deposited seeds of typical riparian plant species by winter floods.

## Supporting information

S1 File Methodological details. (PDF)

S2 File Overview of deposited seed species. (PDF)

S3 File Number of seeds per species in flooded *versus* non-flooded seed traps. (PDF)

S4 File Multivariate analyses. (PDF)

S5 File Seed deposition patterns within the flooded seed traps. (PDF)
