# Supplementary material for: Spatial patterns of water-dispersed seed deposition along stream riparian gradients
Source: PLoS One. 2017 Sep 28;12(9):e0185247. doi: 10.1371/journal.pone.0185247 (PMC5619765; doi:10.1371/journal.pone.0185247)
Supplement: S1 File — (PDF) [file pone.0185247.s001.pdf]

Appendix S1. Methodological details

S1.1. Research locations, additional information

The study was performed at three lowland streams in the Netherlands (Fig S1.1). All streams had been subject to restoration measures along 0.8–2-km stretches of the stream (as described in [1]). Restoration activities at the Hagmolenbeek (HM) were completed in June 2010, at the Hooge Raam (HR) in July 2009 (only the channel) and April 2011 (the riparian zone), and at the Kleine Aa (KA) in July 2011. Transversal profiles after restoration differed between the streams, with channel widths/depths of 2.0/0.3, 7.0/0.2, and 7.0/0.8 m, and riparian zones widths of 25, 15, and 10 m for HM, HR, and KA, respectively. Hydrological conditions varied accordingly, with average hourly discharges of 0.12, 0.15, and 0.85 m^3/s, peak discharges of 1.03, 1.64, and 7.2 m^3/s, and overbank flows amounting to 109, 59, and 139 days, all for the year 2012, except discharge values at KA (March 2013–December 2013). At HM only, the riparian zone was sown with a seed mixture of *Lolium perenne*, *Trifolium repens*, and *Phleum pratense* subsp. *pratense* [2], to reduce possible erosion after restoration.

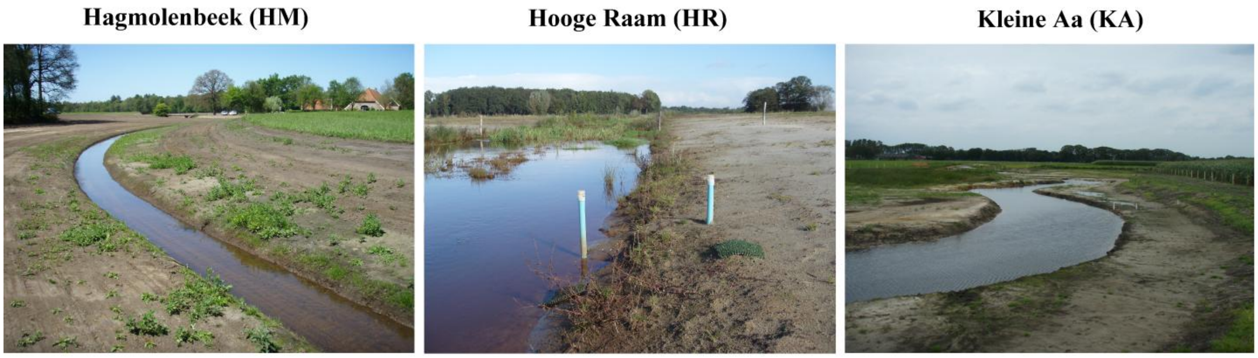

Fig S1.1. Pictures of the three research locations, showing the excavated riparian zones shortly after the restoration measures. From [1].

S1.2. Comparison of seed count methods

Deposited seeds in the seed traps of the HM-site were counted by physically extracting seeds, while for the seed traps of the HR- and KA-sites a seedling emergence method was used. The different seed count methods resulted in similar amounts of species. Only period 3 of the HM-site showed a higher number of species, which was probably caused by the later successional stage, accounting for a more developed seed and species pool in the direct surroundings, compared to the earlier periods (period 1, 2 and 3 represent the first, second and third half year of seed rain monitoring after the stream restoration activities).

Although the total number of seeds was higher for physical extraction in all periods, this difference was largely caused by seeds of three highly abundant species. Without the seeds of these species the total number of seeds per seed trap were much more similar between seed count methods (Table S2.1). This most likely reflected differences between research locations, i.e. a very high abundance of these three species at the HM-site, rather than differences in seed count methods. Overall, these results suggest that the different seed count methods did not greatly affect the results in this study.

Table S1.2. Overview of the number of seeds and species per research location, separately for data including all species and for a subset of the data without the three most abundant species.

All species

Method	Site	Period	Season	Tot nr species	Tot nr seeds	Mean nr species +- sd	Mean nr seeds +- sd
Physical extraction	HM	1	Winter	26	8921	8.3 (2.5)	594.7 (609.4)
Physical extraction	HM	2	Summer	22	3348	8.0 (4.5)	223.2 (254)
Physical extraction	HM	3	Winter	69	3804	16.7 (7.2)	253.6 (299.2)
Seedling emergence	HR	1	Summer	27	380	5.6 (2.3)	25.3 (31.1)
Seedling emergence	HR	2	Winter	33	551	9.3 (2.6)	36.7 (22.6)
Seedling emergence	KA	1	Winter	31	1080	10.0 (2.3)	108 (118.9)
Seedling emergence	KA	2	Summer	27	758	4.3 (2.7)	50.5 (69.3)

Without three most abundant species

Method	Site	Period	Season	Tot nr species	Tot nr seeds	Mean nr species +- sd	Mean nr seeds +- sd
Physical extraction	HM	1	Winter	23	742	6.1 (1.9)	49.5 (29.7)
Physical extraction	HM	2	Summer	20	1576	6.7 (4.4)	105.1 (86.7)
Physical extraction	HM	3	Winter	67	1823	15.4 (7.1)	121.5 (164)
Seedling emergence	HR	1	Summer	25	370	5.0 (2.2)	24.7 (31.3)
Seedling emergence	HR	2	Winter	31	533	8.5 (2.2)	35.5 (21.7)
Seedling emergence	KA	1	Winter	29	569	8.2 (2.2)	56.9 (67.9)
Seedling emergence	KA	2	Summer	26	743	4.0 (2.8)	49.5 (68.1)

References

1. Fraaije RGA, ter Braak CJF, Verduyn B, Breeman LBS, Verhoeven JTA, Soons MB. Early plant recruitment stages set the template for the development of vegetation patterns along a hydrological gradient. Funct Ecol. 2015; 29: 971–980.
2. van der Meijden R. Heukels‘ Flora van Nederland Drieëntwintigste Druk. Groningen: Wolters-Noordhoff; 2005.
