# Supplementary material for: Spatial patterns of water-dispersed seed deposition along stream riparian gradients
Source: PLoS One. 2017 Sep 28;12(9):e0185247. doi: 10.1371/journal.pone.0185247 (PMC5619765; doi:10.1371/journal.pone.0185247)
Supplement: S2 File — (PDF) [file pone.0185247.s002.pdf]

Appendix S2. Overview of deposited seed species

Table S2.1. Total number of deposited seeds per species for the Hagmolenbeek (HM), Hooge Raam (HR) and Kleine Aa (KA) sites, separated for each trapping period (p1, p2 or p3).

Species	HM p1	HM p2	HM p3	HR p1	HR p2	KA p1	KA p2
Achillea millefolium	0	0	1	0	0	0	0
Agrostis capillaris/canina	0	178	1	0	0	0	0
Alisma lanceolatum	0	0	18	0	0	0	0
Alisma plantago-aquatica	0	236	406	24	108	0	137
Allium schoenoprasum	1	0	0	0	0	0	0
Alnus glutinosa	45	1	165	0	0	4	0
Alopecurus geniculatus	0	0	8	0	0	0	0
Angelica sylvestris	1	0	1	0	0	0	0
Atriplex patula	0	0	0	0	1	0	0
Betula pendula/pubescens	465	593	1400	0	0	0	0
Betula pubescens	0	0	0	6	22	29	1
Bidens cernua	0	0	1	0	0	0	0
Bidens frondosa	0	6	25	0	0	0	0
Bidens tripartita	0	0	13	5	6	0	6
Bromus hordeaceus ssp hordeaceus	0	0	1	0	0	0	0
Callitriche platycarpa/obtusangula/stagnalis	0	0	18	12	16	0	0
Capsella bursa-pastoris	42	41	2	0	0	0	0
Carex oederi ssp oederi	0	0	22	0	0	0	0
Carex ovalis	0	0	1	0	0	0	0
Carex pallescens	0	0	1	0	0	0	0
Carex pseudocyperus/rostrata	1	0	5	0	0	0	0
Carex remota	0	0	1	0	0	0	0
Carex riparia	0	0	1	0	0	0	0
Carex sp	0	0	1	0	0	0	0
Cerastium glomeratum	0	0	0	3	0	1	0
Ceratophyllum demersum	0	0	1	0	0	0	0
Chenopodium album	0	0	0	0	0	26	8
Chenopodium album/polyspermum	391	189	239	0	0	0	0
Cirsium arvense	0	0	0	1	3	0	0
Conyza canadensis	0	0	0	18	8	5	71
Conyza sumatrensis	0	3	0	0	0	0	0

Table S2.1 continued

Species	HM p1	HM p2	HM p3	HR p1	HR p2	KA p1	KA p2
Digitaria ischaemum	0	0	0	0	3	0	0
Echinochloa crus-galli	56	69	36	0	0	53	152
Eleocharis palustris	0	0	1	0	0	0	0
Epilobium ciliatum	0	0	0	0	0	46	0
Epilobium tetragonum/ciliatium/parviflorum	0	0	0	30	28	0	0
Euphrasia stricta	0	0	1	0	0	0	0
Fagus sylvatica	0	0	6	0	0	0	0
Festuca brevipilla/rubra/filiformis	0	0	2	0	0	0	0
Festuca pratensis	0	0	1	0	0	0	0
Filipendula ulmaria	0	0	0	0	2	0	0
Galinsoga parviflora	0	0	0	0	1	0	0
Galinsoga quadriradiata	0	0	0	0	0	2	108
Glyceria fluitans	1	8	12	1	0	0	1
Glyceria maxima	0	0	0	5	17	1	22
Gnaphalium luteo-album	0	0	0	5	6	0	0
Gnaphalium uliginosum	853	1179	581	6	10	399	0
*Hieracium sp**	0	1	1	0	0	0	0
Holcus lanatus	1	0	3	0	0	0	0
Holcus mollis	0	0	1	0	0	0	0
*Hypericum sp**	128	0	4	0	0	0	0
Hypericum perforatum	0	0	0	2	24	0	0
Isolepis setacea	7	10	13	0	0	1	0
Jacobea sp	0	0	0	2	0	0	0
Leontodon autumnalis	0	0	0	0	0	0	1
Luzula luzuloides	0	0	1	0	0	0	0
Lycopus europaeus	20	0	36	0	0	192	0
Lythrum salicaria	0	0	0	2	11	4	14
Matricaria chamomilla	0	0	4	0	1	1	0
Matricaria discoidea	0	0	0	0	0	1	0
Mentha aquatica	0	0	0	0	0	27	2
Mentha aquatica x verticillata	0	0	0	0	1	0	0
Myosotis scorpioides	0	0	0	3	6	1	0
Nasturtium microphyllum	0	0	1	0	0	0	0
Oenanthe aquatica	0	0	0	0	0	1	1
Persicaria lapathifolia	7	332	16	0	0	0	0
Persicaria maculosa	1	0	2	152	26	6	0
Persicaria minor	0	12	1	0	0	0	0
Persicaria mitis	0	1	2	0	0	0	0

Table S2.1 continued

**Species**	**HM p1**	**HM p2**	**HM p3**	**HR p1**	**HR p2**	**KA p1**	**KA p2**
Phalaris arundinacea	0	0	1	0	0	0	0
Phragmites australis	0	0	171	0	0	1	1
Plantago major ssp major	0	0	0	9	13	0	0
Poa annua	0	0	4	0	0	0	0
Poa trivialis	0	0	5	0	0	0	0
Polygonum aviculare	2	0	14	1	0	1	0
Potamogeton alpinus/crispus/natans	7	0	3	0	0	0	0
Potentilla anserina	0	0	0	0	0	1	0
*Potentilla sp**	0	0	0	5	2	0	0
Primula vulgaris	0	0	0	0	1	0	1
Ranunculus flammula	0	4	0	0	0	0	0
Ranunculus ololeucos	0	0	1	0	0	0	0
Ranunculus peltatus	0	3	9	0	0	0	0
Ranunculus repens	0	0	0	0	0	1	0
Ranunculus sceleratus	0	148	130	71	76	118	135
Rorippa palustris	6861	0	0	4	8	112	15
Rubus fruticosus	0	0	2	0	0	0	0
Rumex hydrolapathum	0	0	0	0	0	0	1
Rumex obtusifolius	3	93	34	0	1	0	1
Sagina procumbens	0	0	0	3	6	0	0
Salix cinerea	0	0	0	0	0	0	1
Salvia officinalis	0	0	1	0	0	0	0
Sambucus racemosa	0	0	1	0	0	0	0
Scirpus sylvaticus	1	0	0	0	0	0	0
Scrophularia sp	0	0	3	0	0	0	0
Senecio vulgaris	0	0	0	1	7	0	2
Solanum nigrum ssp nigrum	0	0	18	0	13	23	0
Sonchus arvensis	0	0	0	0	0	1	2
Sparganium emersum	2	0	24	0	0	0	0
Sparganium emersum/erectum	0	0	0	0	0	1	49
Sparganium erectum	12	0	32	0	0	0	0
Spergula arvensis	2	0	4	0	0	1	6
Stellaria media	9	7	32	6	18	13	15
Tanacetum vulgare	0	0	1	0	0	0	0
Taraxacum officinale	0	0	11	0	0	0	1
Thuja plicata	0	0	3	0	0	0	0
Trifolium repens	0	0	0	0	0	0	4
Typha latifolia/Sparganium emersum/erectum	0	0	0	2	104	0	0

Table S2.1 continued

Species	HM p1	HM p2	HM p3	HR p1	HR p2	KA p1	KA p2
Urtica dioica	2	0	53	0	1	7	0
Valeriana officinalis	0	0	1	0	0	0	0
Veronica catenata	0	234	189	1	1	0	0

**Hieracium sp: Hieracium umbellatum/laevigatum/sabaudum/vulvatum/pilosella*

**Hypericum sp: Hypericum elodes/humifusum/maculatum/perforatum/tetrapterum*

**Potentilla sp: Potentilla anglica/anserina/argentea/erecta/reptans/sterilis/tabernaemontani*
