# Supplementary material for: Spatial patterns of water-dispersed seed deposition along stream riparian gradients
Source: PLoS One. 2017 Sep 28;12(9):e0185247. doi: 10.1371/journal.pone.0185247 (PMC5619765; doi:10.1371/journal.pone.0185247)
Supplement: S3 File — (PDF) [file pone.0185247.s003.pdf]

Appendix S3. Number of seeds per species in flooded *versus* non-flooded seed traps

Table S3.1. Overview of deposited seeds of species that arrived in both flooded and non-flooded seed traps, with the total number of seeds (subdivided in flooded (FL) and non-flooded (notFL) seed traps) and delta AIC values (dAIC) for the addition of flooding as a fixed effect to Poisson GLMMs explaining the number of seeds deposited. Significant model improvements (dAIC of -2.0 or lower; [1]) are underlined and in bold. Transects were included as random effect (intercept) in all models. Species are ordered in three groups separated by horizontal lines, showing significantly more seeds at flooded seed traps (upper group; positive estimates), non-significantly different numbers at flooded and non-flooded seed traps (center), or significantly more seeds at non-flooded seed traps (lower group; negative estimates).

Species	Tot nr seeds	Nr seeds FL	Nr seeds notFL	dAIC	Estimate
Rorippa palustris	6988	6958	30	**-4142.1**	4.5
Gnaphalium uliginosum	3012	3000	12	**-1399.5**	4.3
Betula pendula/pubescens	2458	2415	43	**-869.3**	2.7
Alisma plantago-aquatica	779	774	5	**-451.9**	4.1
Ranunculus sceleratus	531	510	21	**-178.4**	2.1
Alnus glutinosa	215	211	4	**-77.9**	2.7
Agrostis capillaris/canina	179	166	13	**-24**	1.2
*Hypericum sp**	132	121	11	**-14.2**	1.1
Rumex obtusifolius	131	121	10	**-15.1**	1.2
Urtica dioica	62	59	3	**-20**	2
Epilobium ciliatum	46	42	4	**-2.9**	1
Betula pubescens	30	28	2	**-4.2**	1.5
Glyceria maxima	23	22	1	**-6.8**	2.2
Chenopodium album/polyspermum	819	614	205	0.8	-0.1
Echinochloa crus-galli	366	246	120	-0.2	-0.2
Capsella bursa-pastoris	85	69	16	1.5	0.2
Sparganium erectum	44	31	13	1.1	-0.3
Mentha aquatica	29	16	13	1.5	-0.3
Sparganium emersum	26	23	3	-0.3	0.8

Table S3.1 continued

Species	Tot nr seeds	Nr seeds FL	Nr seeds notFL	dAIC	Estimate
Glyceria fluitans	22	18	4	1.6	0.3
Bidens tripartita	19	13	6	1.1	-0.5
Polygonum aviculare	17	14	3	1.3	0.5
Spergula arvensis	13	7	6	0.1	-0.8
Taraxacum officinale	12	6	6	-0.7	-1
Potamogeton alpinus/crispus/natans	10	6	4	0.6	-0.8
Persicaria maculosa	9	8	1	1.4	0.8
Holcus lanatus	4	2	2	0.7	-1.2
Sonchus arvensis	3	2	1	1.8	-0.5
Persicaria mitis	3	2	1	1.8	-0.5
Rubus fruticosus	2	1	1	1.5	-1
Oenanthe aquatica	2	1	1	1.7	-0.8
Lycopus europaeus	248	89	159	**-157.4**	-1.6
Galinsoga quadriradiata	110	2	108	**-326.4**	-5.4
Conyza canadensis	76	32	44	**-172.2**	-3.9
Stellaria media	76	5	71	**-33.7**	-1.4
Solanum nigrum ssp nigrum	41	16	25	**-14.2**	-1.3
Chenopodium album	34	11	23	**-13.4**	-1.4
Lythrum salicaria	18	8	10	**-4.6**	-1.3

**Hypericum sp: Hypericum elodes/humifusum/maculatum/perforatum/tetrapterum*

References

1. Burnham KP, Anderson DR. Model selection and multimodel inference: a practical information-theoretic approach. New York, NY.: Springer; 2002.
