# Supplementary material for: Spatial patterns of water-dispersed seed deposition along stream riparian gradients
Source: PLoS One. 2017 Sep 28;12(9):e0185247. doi: 10.1371/journal.pone.0185247 (PMC5619765; doi:10.1371/journal.pone.0185247)
Supplement: S4 File — (PDF) [file pone.0185247.s004.pdf]

Appendix S4. Multivariate analyses

S4.1. Deposited seed community composition of flooded *versus* non-flooded seed traps

Table S4.1.1. Overview of conditional effects (flooding and season) in partial redundancy analyses on the number of deposited seeds (Hellinger-transformed), separately per research location (only for the HM and KA-sites, as there were no non-flooded seed traps at the HR-site).

HM site: Variable	Explains %	F	P
Flooding	11.8	7.5	0.001
Season	6.7	4.6	0.001
**KA site: Variable**	**Explains %**	**F**	**P**
Season	12.7	4.5	0.001
Flooding	3.2	1.8	0.033

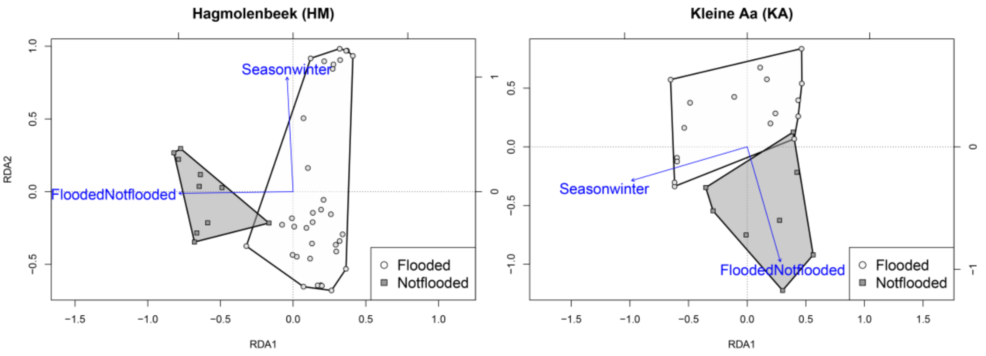

Fig S4.1.1. Distance triplots of partial redundancy analyses on Hellinger-transformed seed deposition per research location with respect to the explanatory variables season and flooding. Site scores are indicated by symbols, with different symbol type for flooded versus non-flooded seed traps. Contour lines are added around sites scores with the same symbol type.

S4.2. Effect of seed trap elevation on the deposited seed community composition along the riparian gradient, using all seed traps

Table S4.2.1. Overview of conditional effects (seed trap elevation and season) in partial redundancy analyses on the number of deposited seeds (Hellinger-transformed), separately per research location (HM, HR and KA-site respectively), using the combined data of flooded and non-flooded seed traps.

HM site: Variable	Explains %	F	P
Elevation	13.6	8.6	0.001
Season	6.8	4.7	0.001

HR site: Variable	Explains %	F	P
Elevation	4.9	3.0	0.001
Season	4.9	2.8	0.003

KA site: Variable	Explains %	F	P
Season	12.7	2.0	0.001
Elevation	4.1	4.6	0.022

S4.3. Effect of seed trap elevation on the deposited seed community composition along the riparian gradient, using only the flooded seed traps

Table S4.3.1. Overview of conditional effects (seed trap elevation and season) in partial redundancy analyses on the number of deposited seeds (Hellinger-transformed), separately per research location (HM, HR and KA-site respectively), using the data of only the flooded seed traps.

HM site: Variable	Explains %	F	P
Elevation	13.9	7.7	0.001
Season	10.2	5.7	0.002

HR site: Variable	Explains %	F	P
Elevation	4.9	3.0	0.001
Season	4.9	2.8	0.003

KA site: Variable	Explains %	F	P
Season	20.4	4.7	0.001
Elevation	-0.2	1.0	0.465

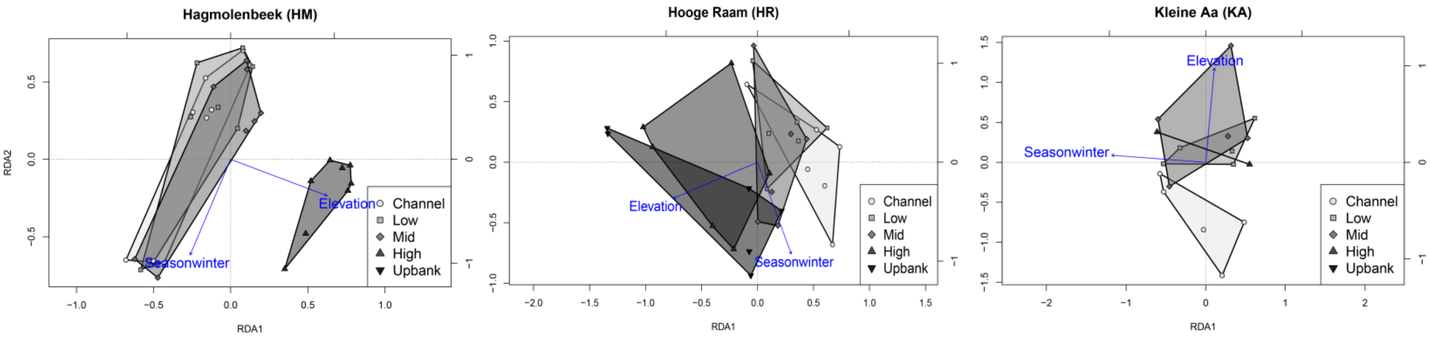

Fig S4.3.1. Distance triplots of partial redundancy analyses on Hellinger-transformed seed deposition per research location with respect to the explanatory variables season and seed trap elevation, using the subset of only the flooded seed traps. Site scores are indicated by symbols, with symbol type determined

by seed trap position along the riparian gradient, ranging from the dry end (upbank) to the wet end (channel) of the riparian gradient. Contour lines are added around sites scores with the same symbol type. Upbank traps are missing in the triplots for the HM and KA-sites as these traps were not flooded.
