# Supplementary material for: Spatial patterns of water-dispersed seed deposition along stream riparian gradients
Source: PLoS One. 2017 Sep 28;12(9):e0185247. doi: 10.1371/journal.pone.0185247 (PMC5619765; doi:10.1371/journal.pone.0185247)
Supplement: S5 File — (PDF) [file pone.0185247.s005.pdf]

Appendix S5. Seed deposition patterns within the flooded seed traps

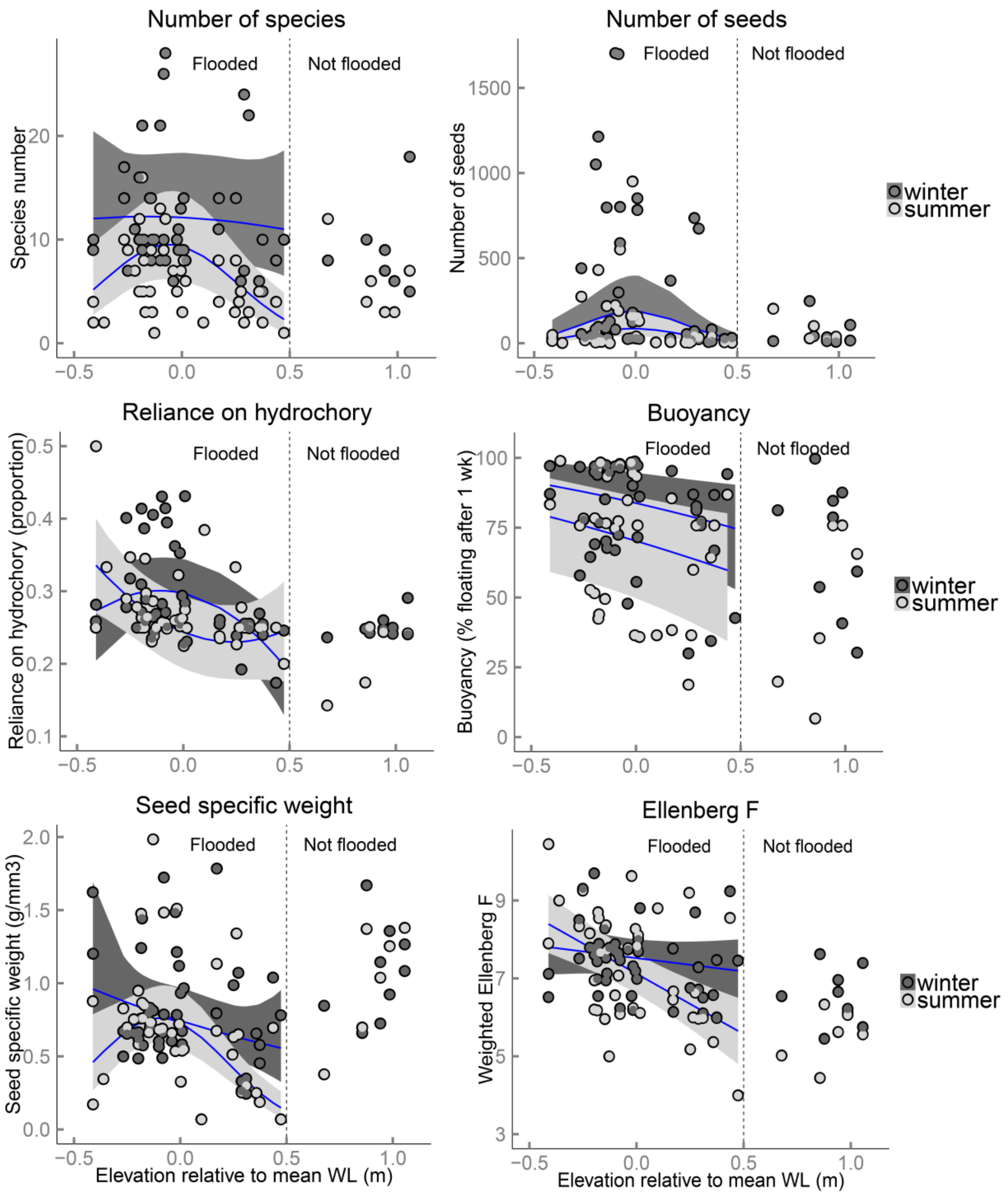

Fig S5. The effects of elevation and season on community-weighted means of seed traits of the deposited seed community, i.e. reliance on hydrochory, seed buoyancy, seed specific weight and Ellenberg F-value. The effect of seed trap elevation relative to the mean water level is given (negative values for seed traps below the average water level, and positive values for seed traps above it). Summer and winter data are displayed by different greyscales. Blue lines represent modeled relationships between the flooded field data (circles) and the explanatory variables season and elevation, as analyzed in LMMs. Grey ribbons indicate the 95% confidence intervals (based on fixed effects only). Non-flooded plot data are shown as reference only.
